# 

**Published:** 1885-12

**Authors:** 


					﻿OLD SERIES
Vol. xxv

WeIIKAL’<UBW’
F JOURNAKgS.
C^pl *1 I'	j?—JZt■ S). j '
r z'7^by“ w.ewestmorelandW.®'
VlA h.v.m.miller.m.d.ll.d,J«l
Lrp	JAMES. AGRAY. M.D.">
C^uBLISHED By"JAMESRHwisON3.CQ^
tcP____________Atlanta , ga. ma
RURSCRIPTION: $2.50 A YEAR.
				

